# Participants' Experiences of, and Views About, Daytime Use of a Day-and-Night Hybrid Closed-Loop System in Real Life Settings: Longitudinal Qualitative Study

**DOI:** 10.1089/dia.2018.0306

**Published:** 2019-03-01

**Authors:** Julia Lawton, Maxine Blackburn, David Rankin, Janet M. Allen, Fiona M. Campbell, Lalantha Leelarathna, Martin Tauschmann, Hood Thabit, Malgorzata E. Wilinska, Daniela Elleri, Roman Hovorka

**Affiliations:** ^1^ Usher Institute of Population Health Sciences and Informatics, University of Edinburgh, Edinburgh, United Kingdom.; ^2^ Wellcome Trust-MRC Institute of Metabolic Science, University of Cambridge, Cambridge, United Kingdom.; ^3^ Department of Pediatrics, University of Cambridge, Cambridge, United Kingdom.; ^4^ Leeds Children's Hospital, Leeds, United Kingdom.; ^5^ Manchester Diabetes Center, Manchester Academic Health Science Center, Manchester University NHS Foundation Trust, University of Manchester, Manchester, United Kingdom.; ^6^ Royal Hospital for Sick Children, Edinburgh, United Kingdom.

**Keywords:** Type 1 diabetes, Closed-loop system, Artificial pancreas, Medical device, User experience, Qualitative research

## Abstract

***Objective:*** To explore individuals' experiences of daytime use of a day-and-night hybrid closed-loop system, their information and support needs, and their views about how future systems could be improved.

***Research Design and Methods:*** Twenty-four adults, adolescents, and parents were interviewed before using a hybrid day-and-night closed-loop system and 3 months later, data were analyzed thematically.

***Results:*** Participants praised the closed loop's ability to respond to high and low blood glucose in ways which extended beyond their own capabilities and to act as a safety net and mop up errors, such as when a mealtime bolus was forgotten or unplanned activity was undertaken. Participants also described feeling less burdened by diabetes as a consequence and more able to lead flexible, spontaneous lives. Contrary to their initial expectations, and after trust in the system had been established, most individuals wanted opportunities to collaborate with the closed loop to optimize its effectiveness. Such individuals expressed a need to communicate information, such as when routines changed or to indicate different intensities of physical activity. While individuals valued frequent contact with staff in the initial month of use, most felt that their long-term support needs would be no greater than when using an insulin pump.

***Conclusions:*** While participants reported substantial benefits to using the closed loop during the day, they also identified ways in which the technology could be refined and education and training tailored to optimize effective use. Our findings suggest that mainstreaming this technology will not necessarily lead to increased demands on clinical staff.

## Introduction

A closed-loop system combines a real-time continuous glucose monitor (CGM) with an insulin pump and an algorithm which translates, in real time, information from the CGM and computes the amount of insulin delivered by the pump. Closed-loop systems require varying levels of user input; for example, to calibrate the CGM and enter information about carbohydrate consumption. To improve usability and acceptability, studies have elicited a user perspective.^1–3^ Many have consulted individuals who have no direct experience of using the technology^1,3–6^ and have found that, as well as having high expectations, people question whether they will be able to trust the system.^3–5^ This has prompted concerns that people's attitudes and expectations may present barriers to using the technology.^5^ When studies have involved individuals who have used the technology, they have mostly focused on overnight systems^7–12^ and have found that participants generally perceive glycemic and other benefits as outweighing logistical burdens.^7–10,12^ However, findings cannot be extrapolated to 24-h systems because insulin requirements and routines are more stable and predictable overnight; this has prompted suggestions that it may be easier to adapt to and trust overnight systems.^7^

Limited research has explored people's experiences of using 24-h systems. Studies have mostly been of very short duration (3–5 days) with high levels of staff monitoring.^2,13,14^ Hence, there have been calls for longer studies to be undertaken in “real world” settings.^2,4,13^ One exception is a mixed-methods study involving adults, children, and adolescents who used a closed-loop system for 12 weeks; although only adults used the system during the day.^15^ While this study offers useful insights, there was limited focus on daytime use, and data reporting mostly cohered around perceived benefits and burdens.^15^

Despite significant advances in the technology, there remains limited understanding of how people engage with, and use, 24-h systems in everyday life and over time. This information is vital for understanding whether, and how, trust in the system develops; what information and support individuals may need to make effective use of the technology^8^; and, how systems might be further improved to meet users' needs. To address these objectives, we report findings from interviews undertaken with individuals who used a hybrid day-and-night closed-loop system combined with pump suspend feature over 3 months. Given that studies have overwhelmingly focused upon nighttime use, we focus our reporting on people's experiences of using the closed-loop system during the day.

## Methods

Qualitative approaches are recommended when little is known about the area under investigation. This is because they allow findings to emerge from the data rather than testing predetermined hypotheses; indeed, qualitative approaches are used to open up new areas of enquiry and generate hypotheses, which can then be tested quantitatively for this reason.^16,17^ In this study, in-depth interviews informed by topic guides were used as these enabled the discussion to remain relevant to addressing the study objectives, while affording flexibility for participants to raise and discuss issues they perceived as salient, including those unanticipated at the beginning of the study.^18^ Interviews also afforded privacy so that participants could talk about sensitive topics (e.g., criticisms of the support received during the trial) should they choose to do so. Topic guides were developed in light of literature reviews, input from patient representatives, and clinical coinvestigators (see Table 1 for information about the main areas explored in the interviews and how the topic guides were used to facilitate discussion with participants). Data collection and analysis took place concurrently so that findings identified in early interviews could be used to inform the topics explored in later ones.

**Table 1.** Key Areas Explored in Baseline and Follow-up Interviews

Baseline interview
• Experiences of diabetes self-management before the trial
• Perceptions and understandings of the closed-loop system, including
○ How it would work
○ How it would impact on diabetes self-management practices
○ Potential impact on blood glucose control
• Any worries and concerns about using a closed-loop system and the reasons for these
• Motivations and agendas for trial participation
Follow-up interview
• Initial experiences of using, and adapting to, the closed loop
• Confidence and trust in the system (was this established, if so why or why not?); reasons for disconnecting or overriding the system
• Impact of using the closed-loop system on everyday work/school and family life
○ Dietary choices
○ Physical activity
○ Approaches to managing and/or preventing hypoglycemia and hyperglycemia
○ Self-concepts and relationships with others ○ worries and concerns about having diabetes
• Views about how the closed loop could be improved to aid efficacy and acceptability
• Views about the training and support received during the trial (e.g., was it sufficient; how could it be improved?)
• Longer-term support needed to use the closed loop in routine clinical care

While the same general areas, outlined above, were covered with all participants, tailored questions were also asked and probes used to encourage and enable a fuller elicitation of responses to particular questions. We also tailored some of the questions asked in each participant's follow-up interview to take account of the kinds of information and experiences they had shared in their baseline interview.

### Recruitment and data collection

Participants were recruited following randomization to a 3-month clinical trial (APCam11) designed to assess the effectiveness of a hybrid day-and-night closed-loop system combined with pump suspend feature compared with sensor-augmented pump therapy (control arm).^19^ For more detail about the closed-loop system and training and contact received during the trial see Table 2. To be eligible for this trial, individuals had to have used an insulin pump for ≥3 months, a screening HbA1c ≥7.5% (58.5 mmol/mol) and ≤10% (86 mmol/mol) and a diabetes duration of ≥6 months.^19^

**Table 2.** Description of the Closed-Loop System and Trial Procedures

The FlorenceM closed-loop system utilized a model predictive control algorithm (version 0.3.46; University of Cambridge, Cambridge, United Kingdom) residing on a smartphone (Galaxy S4; Samsung, South Korea). Every 10 minutes, the control algorithm calculated an insulin infusion rate, which was set on the study pump. The control algorithm was initialized using preprogrammed basal insulin delivery downloaded from the study pump. Information about the participant's weight and total daily insulin dose were entered at setup. The treat-to-target control algorithm aimed to achieve glucose levels between 5.8 and 7.3 mmol/L depending on the accuracy of model-based glucose predictions.
Training and staff contact received during the trial:
The study included up to 11 visits and six telephone/email contacts for subjects completing the study. Participants randomized to the closed-loop group attended the clinical research facility/usual clinic for a 2- to 3-h visit. Training was provided on initiation and discontinuation of the hybrid closed-loop system, switching between closed loop and standard insulin pump therapy, meal bolus procedure, and the use of study devices during exercise. A closed-loop system user manual, including a trouble-shooting section, was handed out during the initial training session. Competency on the use of the closed-loop system was assessed. Participants were contacted within 24 to 48 h after the initiation of study treatment. During the first 2 weeks of the intervention, participants were contacted (the United Kingdom) or seen (the United States) in the clinic weekly. Thereafter, participants were contacted monthly. All participants were provided with a 24-h helpline to contact the study team in the event of study-related issues.

Individuals randomized to a closed loop were invited to opt-in to the interview study by staff in the four participating U.K. sites. Participants comprised individuals ≥16 years of age, individuals 13–15 years of age and their parent(s), and parents of those ≤12 years of age; all individuals approached agreed to take part. Recruitment continued until there was adequate representation of different age groups and data saturation had occurred; that is, until no new findings were identified in new data collected. The study received approval in the United Kingdom from Cambridge East Research Ethics Committee (REC ref 15/EE/0324) and the Medicines & Health Products Regulatory Agency.

Participants were interviewed before commencing use of the closed-loop system (baseline) and 3 months later. This design permitted initial expectations to be compared with actual experiences of using the technology. Interviews were conducted by M.B., an experienced qualitative researcher who was not a member of the clinical team, at a time and location of participants' choosing (mostly in their own homes). Interviews averaged 1–2 h, and were transcribed in full. Before interviews were undertaken, it was made clear to participants that the qualitative research team was independent of the trial team and that all information shared would be kept strictly confidential.

### Data analysis

Interviews were analyzed by J.L. (an experienced qualitative researcher) and M.B. using a thematic approach informed by the method of constant comparison. Interviews were cross-compared to identify recurrent themes^20^; we also explored differences and similarities in participants' experiences across different age groups and over time by comparing baseline and follow-up accounts. A coding framework was then developed by J.L. and M.B., which captured key findings and themes and contextual information needed to aid data interpretation. Nvivo, a qualitative software package, was used to facilitate data coding and retrieval and coded datasets were subjected to further analyses to allow more nuanced interpretations of the data to be developed.

## Results

The sample comprised 10 adults (≥18 years of age), 5 adolescents (13–17 years of age) and 9 parents. Interviewee demographic data are presented in Table 3.

**Table 3.** Demographic Characteristics of Study Participants

Participants with type 1 diabetes (*n* = 15)	
Gender, female, *n* (%)	7 (46.7)
Age at recruitment (years)	
13–17	5
18–30	1
31–40	6
41–50	2
51–60	
60+	1
Occupation/education, *n* (%)	
Professional	5 (33.3)
Semiskilled	4 (26.7)
Retired	1 (6.7)
Higher education	2 (13.3)
Secondary school	3 (20)
Previous involvement in closed-loop system trial(s)	6 (40)
Parents of pediatric patients (*n* = 9)^a^	
Gender, female, *n* (%)	7 (77.8)
Age at recruitment (years)	
31–40	2
41–50	5
51–60	2
Occupation, *n* (%)	
Professional	5 (55.6)
Semiskilled	3 (33.3)
Unemployed/full-time carer	1 (11.1)
Child had previous involvement in closed-loop system trial(s)	3 (33.3)

^a^ This includes parents who represented children ≤12 years of age (*n* = 5) and parents of children 13–15 years of age (*n* = 4). In one instance, both parents of a child 13–15 years of age participated in an interview.

Below, we begin by reporting participants' initial expectations of the closed-loop system before describing their actual experiences of using this technology in everyday life; this includes exploration of the perceived clinical and quality-of-life benefits of using a closed loop and how, and why, participants wanted to continue to take an active role in diabetes management by collaborating with the system. We conclude by reporting participants' views about the training and support received during the trial and their opinions about the support needed to use a closed loop in the longer-term. As all the main findings cut across the sample, our reporting has not been separated out according to participant groups (e.g., adults, adolescents, and parents); however, we do indicate when a particular issue was most keenly felt within one particular group.

### Initial expectations (baseline interviews)

#### Better and more stable glycemic control

At baseline, all participants conveyed an understanding, informed by previous trial participation and/or reading about the technology (e.g., in newspapers or online), that the closed loop would help regulate blood glucose by bringing it down when it started to go high and suspending or reducing the basal rate when blood glucose started to drop: “It's aiming to get rid of the peaks and troughs, or to narrow the peaks and troughs as much as possible” (Adult#4). Most also suggested that the closed-loop would be better equipped to address high rather than low blood glucose because, while it could administer insulin to lower blood glucose, once insulin was in one's system, “it can't suck the insulin back out so if it's like, if you've bolused too much there is that risk still of a hypo” (Adult#8). Hence, while most people envisaged that they would be able to step back and allow the closed loop to regulate blood glucose on their behalf, they also anticipated still needing to look out for, and treat, hypoglycemia. However, many were also hopeful that, because the closed-loop system might detect and respond to blood glucose dropping more quickly than they could, they would experience less severe episodes of hypoglycemia (Table 4).

**Table 4.** Participant Quotations

*Theme and subtheme*	*Participant quotations*
Initial expectations (baseline line interviews): Better and more stable glycemic control	“So, if I was doing my cycle ride from here to the station I know that it would switch off before I noticed that it was going down really really quickly and maybe prevent a bigger hypo… it may help sort of prevent the worry of a really big one happening.” (Adult#3)
Using the closed-loop system (follow-up interviews): Developing trust and confidence in the system	“it has like a 3 day memory on it. So if you changed to like when he went on holiday- in the school holidays- it took the artificial pancreas a long time to readjust…. Before I didn't understand why he was going hypo, so I was trying to interfere. As the trial got on and went further, I understood its mindset- and- I understood what it was doing, and I could deal with it better, if that makes sense… the more I understood it, the more I could work with it, and the more I could trust it.” (Parent#8)
Clinical and quality-of-life benefits: A more flexible and active life	“it probably has helped a lot because, as far as my sugar levels were concerned, eating and anything else, I could get away with putting it off a little bit longer, because I knew that if my sugar levels were starting to go down a little bit the closed-loop system could deal with that.? So I could put off having something to eat for an extra 30 minutes or an extra 60 minutes. So in that regards yeah, it was a lot better for me personally.” (Adult#1)
	“if I've gone for a bit of unplanned exercise, or I've been a bit more active than I would normally be I've not really logged it as exercise, then the closed-loop drops the insulin, the basal flow, to account for that, which then keeps the blood sugar back in range again. And otherwise, if I'd hadn't remembered to change my basal rate, I'd have had a low.” (Adult#4)
Views about education and training and need for staff support: Training and support needs during the 3-month trial	“I wouldn't have probably learnt as much about it as I did if I hadn't been able to question them. If they'd told me everything that I've learnt on the first week or two, I would have forgotten it by now. By actually being in contact with them and learning it as I've been going along, I've maintained the information a lot more… [because] you don't necessarily remember it until you've actually experienced or you've drilled it in. It's like going through university. You go to your lecture. And unless you go and do a bit more research you forget most of it.” (Adult#9)
	“It's learning how to use all the features properly. But also maintaining a memory of what the different features are because some people are not going to require the exercise feature at all, whereas I needed to use it on a daily basis to take the dog for a walk and first thing in the morning for work… That was all- all those things we figured out within the first couple of weeks. So I learnt that those features were required.” (Adult#1)

#### Probationary period

Despite having positive expectations, participants described feeling ambivalent about handing control over to the closed loop and speculated that, in the initial days and weeks of use, they would be checking their blood glucose regularly and disconnecting or overriding the system if they had any doubts about how it was functioning. Parents also noted how they would be keeping their child under close supervision during this time: “I'm absolutely not gonna let him out of my sight… I suppose its trust, you know, it's not just a broken leg. It's his life. And it's delivering deadly insulin into my son, 24/7.” (Parent#2)

### Using the closed-loop system (follow-up interviews)

#### Developing trust and confidence in the system

In keeping with their initial expectations, participants, at follow-up, described an initial probationary period, typically lasting several weeks, when they had scrutinized the closed-loop system's graphs frequently to seek reassurance that it was working in safe and effective ways: “Certainly the first week, I was looking at the graphs sort of every five minutes (laughing) saying: what's it doing now. And I think after a week of that, I was like: oh well, you know, I was still looking at it frequently but less so.” (Adult#10)

Participants also described how, as a result of seeing the closed loop making logical and sensible decisions, and responding to high/low blood glucose faster than they could that, over time, they had felt more confident and able to allow it to operate without constantly scrutinizing its actions: “You can see it doing its job, and you can see how it's doing it. And I'll be honest, I could see that it was doing a better job than I could do myself.” (Adult#9)

Some individuals also described this initial adjustment period as a two-way process wherein, while they were scrutinizing the system's functioning, the closed loop was calibrating itself to their specific insulin requirements. During this calibration period, some also noted how, in hindsight, their own anxieties and resultant propensity to take action to address high/low blood glucose might have been counterproductive to the system's learning. This included Adult#10 who described how, initially, “I did do my own correction if I felt it wasn't doing it fast enough” until they realized that, “it wasn't going to be helpful for it to work out what I actually needed to be given for the corrections” and Parent#7 who reported how:

“obviously the artificial pancreas recognizes after a few days his patterns. But I was interfering, because I was thinking: oh my gosh, he's going into hypo, this is crazy… And actually when I stopped doing stuff and I allowed the artificial pancreas to do its thing, it became a lot better.”

Some participants, especially parents or those whose jobs or other commitments meant that they did not follow routines, noted that their confidence in the system only developed after they had gained a full appreciation of how the algorithm worked; specifically that it learnt insulin requirements by monitoring and using blood glucose data acquired over several days of previous use. These individuals discussed how, once this understanding had been established, they had better appreciated why blood glucose control could be thrown out on atypical days; hence, they no longer worried that the system was malfunctioning or tried to intervene to correct or override it (Table 4).

### Clinical and quality-of-life benefits

#### Lessening the burden of self-management

Participants reported how using this technology had lessened the burden of diabetes management as they had needed to do finger-prick checks and administer corrective doses less frequently. This, as participants further noted, had had a positive impact on how they felt about having diabetes wherein: “it made me not really care that I am diabetic” (Adolescent#4) or, “it's definitely made a material difference to the amount of time I've had to think about being a diabetic” (Adult#4). Parents also noted an improvement in relationships with their children because there had been less need to constantly remind them to undertake self-management tasks, such as blood glucose testing.

#### Better blood glucose control

Participants also pointed to the clinical and quality-of-life benefits of having, “somebody else watching out for me, in the sense that I could have corrections made to my blood sugars without me being aware” (Adult#10). Indeed, in line with their initial expectations, participants reported experiencing better and more stable blood glucose. This, as participants further noted, was because the closed-loop system had been able to provide a level of responsiveness which had extended beyond their own human capabilities, given the difficulties of keeping blood glucose levels and insulin requirements under constant scrutiny, especially when they were working or distracted by other responsibilities during the day: “I'm a single parent and I have to go to work. So I can't live and breathe diabetes every single day, every single minute of every hour. I don't have the time. But the artificial pancreas did.” (Parent#7)

#### Reassurance and less worry

Participants also noted gaining reassurance from the closed loop being able to “mop up” errors, such as when they had miscounted the carbohydrate content of meals or forgotten to administer insulin before eating: “if I forget to bolus I don't have- I've not got anything covering my back. I've just got to do what I think's right, whereas the [closed-loop] actually made a lot of the smart decisions for you” (Adult#9). This unanticipated benefit of using a closed-loop system was particularly highlighted by teenagers and their parents who noted how it had provided a safety net at a point in the life course when there was greater potential for self-management to be neglected:

“say I was staying round a friend's house, cause my mum worries quite a lot. And like she said that she never really had to, because she just knew this phone would be sorting me out like, say I forgot to give myself insulin like it would end up—like yeah, sorting me out. And everything would like just be fine.” (Adolescent#4)

Participants also noted the additional reassurance gained from their knowledge that, if blood glucose started to drop too low, such as after unplanned physical activities had been undertaken, “I've got the back-up that the closed loop would have turned the insulin off” (Adult#2). However, some participants with poor awareness of hypoglycemia and parents of young children noted that they had needed the pump suspend feature to be set to a higher threshold to achieve this sense of hypoglycemic safety.

#### A more flexible and active life

Due to the level of responsiveness and perceived hypoglycemic safety offered by the closed loop, participants noted how this technology had permitted and enabled a more flexible and spontaneous life. This included no longer having to stick to regular mealtimes, as Adult#1 noted, or as Adult#4 described, having to try to remember to change the pump's basal settings to mitigate risk of hypoglycemia when undertaking moderate physical activities (Table 4).

Such benefits were particularly noted by parents of younger children who reported how they had felt, able to give their child more freedom (e.g., to play with friends, go on school trips, and attend sleepovers) because both their own and other people's (e.g., teachers') confidence in the child's safety had been increased:

“because of the loop we even allowed her to go on a residential holiday with the school for a week. So the teacher was confident as well. Yeah, it was good … She could participate in every single activity at school. She was invited to go to sleepovers, because people felt confident that she's gonna be fine… We could even go out with [friends] as people were happy to babysit. So yes, we had lots of freedom and confidence.” (Parent#4)

### Working in partnership with the closed loop

#### Collaborating with the closed loop to optimize blood glucose control

While most had anticipated taking a back seat and allowing the closed loop to regulate blood glucose on their behalf, only a minority found they had done so in practice. Most reported how they had been prompted to continue to take an active role in glycemic management by the extra data to which they had easy access as a result of using the system: “In some ways you think about it less, cause you think: oh it's all just being taken care of. But then in some ways you think about it more because that information is there to hand” (Adult#6). Specifically, participants discussed how, on occasions, and in light of the CGM alerting them to blood glucose dropping/rising, they had chosen to collaborate with the closed loop. Most typically, this collaboration had occurred when participants had been alerted to high blood glucose, had realized this had resulted from an error (e.g., carbohydrate miscalculation) on their part and had chosen to administer a corrective dose because the closed loop “couldn't cope with the speed of the rise” as Adult#7 noted and as Adult#4 similarly reported:

“So if I can see from the graph my blood glucose increasing, I'd think: right okay. Oh yeah, I've had a cream cake or I've had a biscuit or something I haven't accounted for. So I'll just give it a- I'll do- based on- based on the bolus wizard—how much insulin it thinks I need based on that. And I'll take that then, knowing that the closed-loop will account for what insulin's on board. So it just speeds up the correction a little bit.” (Adult#4)

#### Wanting opportunities to communicate information to the closed loop

Participants also emphasized the benefits of being able to understand how the closed-loop system worked to work with it to increase its effectiveness. To this end, while being praiseworthy of the system's ability to learn insulin requirements based on previous days of learning, some highlighted a need to communicate information about plans and intentions on atypical days, such as weekends or holidays: “it'd be much better if I could press a button and say: I'm really busy today. Don't be so harsh. Or, I'm having a really lazy day, you know, brush it up a bit.” (Adult#5)

In addition, while liking being able to announce planned physical activity to the closed loop, some participants suggested that the efficacy of future systems might be improved by allowing users to discriminate between different intensities of activity.

“There was a function where you could say: I'm going to be doing activity for the next half an hour or whatever… but I couldn't choose the intensity. So I couldn't sort of say, you know…I'm doing high intensity activity. Or I'm just gonna be walking for the next half an hour, which is quite different to how it affects your blood sugars.” (Adult#5)

### Views about education and training and need for staff support

#### Training and support needs during the 3-month trial

In general, participants described the closed-loop system as having been intuitive, self-explanatory, and easy to use: “it was a very easy system to pick up, it was, you know, calibrate the sensor and press start” (Adult#1). Hence, most suggested that an initial training session had been sufficient to feel able to start using the system, especially as they had written materials to refer back to (Table 2).

While initial training was valued, some described how they had not taken in or retained key information, as its relevance had only become apparent after they had started using the closed loop, such as when they first needed to do high-intensity activity. These individuals highlighted the benefits of receiving tailored education and trouble-shooting opportunities with staff, extending over the first month of use, to help make full and effective use of the technology (Table 4).

Participants also described valuing opportunities to contact staff by phone and email in the initial weeks of use. In most cases, this was to resolve technical problems (e.g., system errors), which participants suggested were inevitable given “it's a prototype” (Adolescent#5) and which they believed would be addressed before the system becoming commercially available. Many also described benefitting from glycemic support from staff (e.g., advice about changing mealtime ratios, basal settings); for instance, after noting blood glucose repeatedly going low at a particular time of day.

#### Longer-term support needs

Individuals also noted how their need for staff input had generally declined after the initial month of use and suggested that, were the closed loop to be introduced into routine clinical care, their need for staff support after the initial weeks of use, would be no greater than that required when using a pump:

“I think- the same with any pump that I've been on—when I've moved onto a pump I've always been like given a number to call, or if I've got any questions, speak to this person. And I think for the first couple of months you may have questions. But once you get used to it, you just sort of manage it yourself. So I don't think there'd be any change from the support you get on a closed-loop than you do on a standard pump.” (Adult#9)

## Discussion

By exploring participants' experiences of using a hybrid day-and-night closed-loop system during the day we have shown that the perceived glycemic and quality-of-life benefits of overnight systems^7–10,12^ extend into daytime use. In line with their initial expectations, and main trial results,^21^ participants were particularly praiseworthy of the system's ability to automatically address high and low blood glucose. Other benefits only became apparent after participants had started to use the closed loop. These included the system being able to act as a safety net and mop up errors, such as when mealtime boluses were forgotten, the carbohydrate content of food was miscalculated, or unplanned activity was undertaken. In highlighting these anticipated and unanticipated benefits, participants also noted how using the closed loop had enabled them to feel more confident and normal, less burdened by diabetes, and more able to lead flexible, spontaneous lives.

The benefits of using the closed-loop system were particularly keenly felt by adolescents and parents who suggested that it offered a glycemic safety net at a point in life where diabetes management could be neglected. Indeed, in line with these participants' accounts, clinical research has shown that, in the adolescent age group, a closed-loop system can partly compensate for boluses being forgotten or miscalculated.^22,23^ Parents of young children also noted particular benefits arising from using the closed-loop during the day, including increased confidence in their child's safety and, hence, their being more willing to allow them to participate in activities, such as going on school trips and playing at friends' houses. These findings stand in contrast to those from studies involving parents of children using injection and pump regimens who tend to cocoon and closely supervise their child to help mitigate concerns about severe hypoglycemia, with a detrimental impact on both their own and their child's quality of life.^24^

Participants, at baseline, conveyed realistic expectations of the closed loop, which contrast with findings from earlier studies where participant expectations of the technology were high and sometimes unrealistic.^3–5^ This difference might be due to some of our participants having previously taken part in closed-loop research (Table 3). In addition, there is now much more information about closed-loop systems in the public domain permitting a greater understanding of the technology than would have been the case before closed-loop systems were first developed and trialed. Indeed, participants in our study described having been exposed to this information before the trial.

In keeping with potential users' expectations,^4^ participants reported taking time (typically several weeks) to develop confidence and trust in the closed-loop system. In doing so, participants, like users of overnight systems,^8^ described valuing having easy access to graphical information, which allowed them to observe, and understand, how the system worked. Some also described how, to develop trust, they had needed to know that the closed-loop-based insulin administration on the three previous days of use. Hence, it is vital that this information about how the closed-loop system works is included and emphasized in initial training. Users would also benefit from knowing that the system takes several days to calibrate to their insulin requirements to help avoid panic, worry, and/or their taking action which might compromise its learning.

As well as finding individuals take longer to adapt to 24-h systems than studies of shorter (≤5 days) duration have suggested,^2,13,14^ our findings challenge assumptions that, once trust has developed, users will step back and hand over control to the system.^1,14^ Contrary to this assumption, most participants described preferring to collaborate with the closed loop; for instance, by administering a corrective dose to help bring down high blood glucose. Participants also described wanting opportunities to communicate information to the closed loop, such as when routines changed or a school holiday started. Given these collaborative opportunities applied to daytime use, and were only identified over time, it is unsurprising that they have not been reported in studies involving users of nighttime systems^7–12^ and 24-h systems used for short durations.^2,13,14^

In line with participants' suggestions, systems could be developed which allow users to communicate information about plans and intentions on atypical days and which discriminate between weekdays and weekends. To help prevent hyperglycemic excursions when doing resistance/high-intensity training, and hypoglycemic excursions when doing aerobic/endurance training, systems which allow users to input information about the intensity and type of planned activity could also be considered. “Exercise smart” systems, which automatically receive information about, and respond to differing levels and durations of physical activity, may also help address some users' needs.^25,26^ Individuals who chose to have the pump suspend feature set to a higher threshold to mitigate concerns about hypoglycemia might benefit from systems which administer glucagon.^27,28^

While participants valued initial training and input from staff, including opportunities to contact them to troubleshoot issues, these were mostly to address technical difficulties arising from using a prototype. It is also reassuring that, once initial adjustments had been made, participants felt their need for glycemic support was no greater than that required when using an insulin pump; indeed, their support needs appear very similar to those reported by participants after converting to flexible intensive injection and pump regimens.^29–31^

A key study strength is the use of a qualitative design which enabled identification of issues which have not previously been identified in the literature and which could be further tested and explored in future quantitative research. The 3-month study period permitted in-depth exploration of how people used a 24-h system in everyday life and over time, thereby allowing us to offer a level of insight not previously reported. However, it is possible that, with longer follow-up, participants, like CGM users,^32^ might have experienced technology fatigue or have had changing needs for health professional input. As is typical in closed-loop studies, our sample, which was recruited from a clinical trial, was skewed toward educated and highly motivated individuals,^5,7,9,10,15^ which might partly explain their wish to actively engage with the system. In addition, participants had previous insulin pump experience, and some had prior experience of using a closed-loop system, which might have helped expedite their adjustment to, and use of, the technology in the current study. Hence, future (mixed-methods) studies are recommended. This might include studies of longer duration, involving use of different types of closed-loop systems, undertaken in nontrial (i.e., routine clinical care) settings, and targeting individuals belonging to lower socioeconomic groups. To fully establish users' clinical support needs, future evaluations could also involve quantitative assessment of support solicited and received from clinical staff (e.g., measurement of phone and email contact and frequency of clinic visits).

## Conclusion

Our study has demonstrated how, alongside its clinical benefits,^18^ closed-loop technology can have a very positive and meaningful impact on users' and caregivers' lives by enabling them to feel more normal and less burdened by diabetes. By drawing on participants' perspectives and experiences over time, we have also identified ways in which the technology could be further refined and education and training tailored to optimize effective use.
